# Energy-Density Improvement in Li-Ion Rechargeable Batteries Based on LiCoO_2_ + LiV_3_O_8_ and Graphite + Li-Metal Hybrid Electrodes

**DOI:** 10.3390/ma12122025

**Published:** 2019-06-24

**Authors:** Ki Yoon Bae, Sung Ho Cho, Byung Hyuk Kim, Byung Dae Son, Woo Young Yoon

**Affiliations:** Department of Materials Science and Engineering, Korea University, 1, 5-ga Anam-dong, Sungbuk-gu, Seoul 136-701, Korea; nayehaha@korea.ac.kr (K.Y.B.); s17176@naver.com (S.H.C.); mekiss00@naver.com (B.H.K.); Eric.sohn88@gmail.com (B.D.S.)

**Keywords:** high-energy Li-ion cell, lithium-metal battery, lithium cobalt oxide, lithium trivanadate, lithium-metal powder anode

## Abstract

We developed a novel battery system consisting of a hybrid (LiCoO_2_ + LiV_3_O_8_) cathode in a cell with a hybrid (graphite + Li-metal) anode and compared it with currently used systems. The hybrid cathode was synthesized using various ratios of LiCoO_2_:LiV_3_O_8_, where the 80:20 wt% ratio yielded the best electrochemical performance. The graphite and Li-metal hybrid anode, the composition of which was calculated based on the amount of non-lithiated cathode material (LiV_3_O_8_), was used to synthesize a full cell. With the addition of LiV_3_O_8_, the discharge capacity of the LiCoO_2_ + LiV_3_O_8_ hybrid cathode increased from 142.03 to 182.88 mA h g^−1^ (a 28.76% improvement). The energy density of this cathode also increased significantly, from 545.96 to 629.24 W h kg^−1^ (a 15.21% improvement). The LiCoO_2_ + LiV_3_O_8_ hybrid cathode was characterized through X-ray diffraction analysis, scanning electron microscopy, and energy-dispersive X-ray spectroscopy. Its electrochemical performance was analyzed using a battery-testing system and electrochemical impedance spectroscopy. We expect that optimized synthesis conditions will enable the development of a novel battery system with an increase in energy density and discharge capacity.

## 1. Introduction

The increase in demand for energy devices in various fields has necessitated the development of Li-ion secondary batteries (LiBs). As a cathode material, lithium cobalt oxide (LiCoO_2_; LCO) has played a major role in enabling these applications because of its high nominal voltage, good cycle retention, and structural stability during charging and discharging. However, satisfying the requirements of current electrical devices and energy-storage systems using this active material is difficult because of its low capacity and energy density [1,2]. Therefore, extensive studies have been conducted to improve the low capacity of LCO and the electrochemical performance of LiBs using methods such as doping [3,4,5], surface coating of the LCO [6,7,8,9], and synthesizing composites [10,11,12,13]. However, these methods have not sufficiently improved the low capacity of LCO and are difficult to control because of variable synthesis conditions and different atmospheres, thus resulting in poor reproducibility.

Herein, we introduce a facile, simple, and reproducible method for increasing the capacity and energy density of LiBs by preparing a combined LCO and lithium trivanadate (LiV_3_O_8_; LVO) hybrid cathode. LVO has received considerable attention as a high-capacity cathode material because of its theoretical capacity of approximately 280 m Ah g^−1^, which is more than twice that of LCO. LVO exhibits non-lithiated features and a stable layered structure [14,15,16,17,18]. By using various LCO:LVO constitutional ratios, we determined the optimal composition to achieve enhanced electrochemical performance. To construct the full-cell system, we used Li-metal powder (LP)-deposited graphite as the anode [19,20,21,22,23,24,25]. Because of the non-lithiated characteristics of LVO, the amount of used LVO should be compensated where the application of LP is possible [26,27].

In this study, we report a novel battery system consisting of an LCO + LVO hybrid cathode and a graphite + LP hybrid anode. Because the electrochemical performance of the cathode active materials (LCO and LVO) with respect to their cycle performance and rate capability has been previously characterized [14,15,16,17,18,28,29,30], the main objective of this study was to increase the discharge capacity and energy density during the first 20 cycles. The discharge capacity and energy density of the optimized hybrid cathode were 182.88 mA h g^−1^ and 629.24 W h kg^−1^, respectively, representing enhancements of 28.76% and 15.25%, respectively, as compared with the corresponding values for bare LCO. An improved battery with an increased discharge capacity and energy density was obtained using the proposed strategy.

## 2. Materials and Methods 

A hybrid cathode with LCO and LVO active materials was prepared. The former was obtained from Sigma–Aldrich (Saint Louis, MO, USA) and the latter was synthesized as follows. The LVO precursor was prepared by wet-milling a mixture of LiOH, V_2_O_5_, and ethanol (as a solvent). The milling was performed at room temperature for 6 h, and the mixture was subsequently dried for 24 h under reduced humidity. The resulting ochre powder was heated to 500 °C in an air atmosphere for 10 h in a box furnace. Four hybrid cathode samples were prepared with the following component ratios: LCO10 (LCO 100 wt%), LCO9 (LCO 90 wt%, LVO 10 wt%), LCO8 (LCO 80 wt%, LVO 20 wt%), and LCO7 (LCO 70 wt%, LVO 30 wt%). The hybrid cathodes were prepared by mixing the components in a ball-milling machine for 30 min at 1500 rpm. The cathodes were fabricated by casting slurries containing the active materials (LCO + LVO hybrid cathode; 80 wt%), Ketjenblack (conductive material; 15 wt%), and carboxymethylcellulose (CMC; binder, 5 wt%) onto Al foil. Following the casting, the electrodes were dried at 70 °C for 12 h, and the active mass loading of the electrodes was 2.5 mg cm^−2^ (electrode density: 0.61~0.67 g cm^−2^).

**Figure 1:**
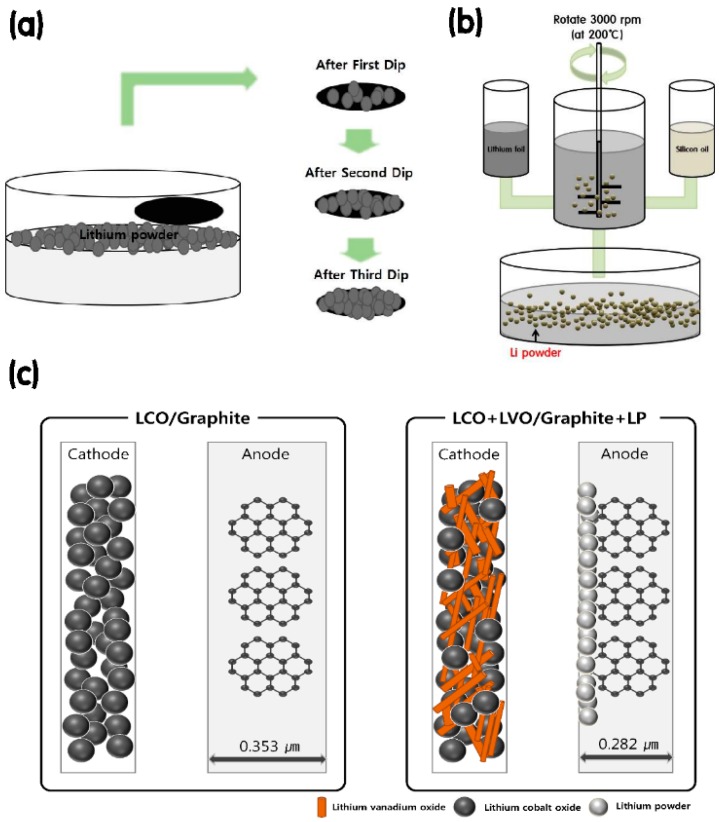
Schematics of (**a**) the dipping method used to coat LP on the graphite anode, (**b**) the LP manufacturing process based on droplet emulsion, and (**c**) the difference in the anode thickness between the LCO10 and LCO8 battery systems.

The graphite + LP hybrid anode was prepared as follows. A graphite electrode was fabricated by casting a slurry containing mesocarbon microbeads (90 wt%), Ketjenblack (5 wt%), and CMC (5 wt%) onto Cu foil, followed by drying at 70 °C for 2 h. The LP layer was formed on the graphite anode surface through dipping in a suspension of LP in dimethyl carbonate, as illustrated in Figure 1a. A pressure of 100 psi was applied to the anode using a vertical hydraulic press to increase the contact between the graphite electrode and LP, which was prepared using the droplet emulsion technique to generate an average size of ≤20 µm [21,22,23,24,25,26,27], as shown in Figure 1b. Table 1 presents the mass percentages of each component in the cathode and anode. The anode mass (graphite + LP) decreased with decreasing graphite content and increasing Li content. The mass of each component was calculated based on the capacity of the LCO + LVO cathode materials. Figure 1c schematically illustrates this effect for the LCO10 and LCO8 samples. The anode thickness was reduced by approximately 25% in the LCO8 system as compared with that in the LCO10 system, thus enhancing the electrochemical performance. Therefore, increasing the anode density using the LP was expected to increase the energy density of the novel battery system. 

The morphologies and structures of the hybrid cathodes and anodes were analyzed using X-ray diffraction (XRD; Rigaku, SmartLab, Tokyo, Japan), scanning electron microscopy (SEM), and energy-dispersive X-ray spectroscopy (EDX; FEI, Quanta 250 FEG, Hillsboro, OR, USA). A CR2032 coin-type cell was assembled for electrochemical analysis, and a Celgard 2500 film was used as the separator. LiPF_6_ (1 M) in a mixture of ethylene carbonate and diethyl carbonate (1:1 *v*/*v*) was used as the electrolyte. After assembly, each cell was aged for 24 h and examined using a battery-testing system (Wonatech Co., Seoul, Korea) at a current density of 0.1 C-rate at 1.8–4.2 V for 20 cycles. This voltage window contained the potential plateaus of both the LCO and LVO. The voltage window of the cell in this experiment is lower than the current trend. However, the industrial feasibility of this electrode system is acceptable in utilization considering a multi-cell pack system application or the next generation LiBs system, such as lithium-sulfur or lithium-air [28,29,30,31]. Electrochemical impedance spectroscopy (Solartron SI1280B, Anyang, Gyeonggi-do, Korea) was performed from 10^−1^ to 10^5^ Hz at 5 mV s^−1^. The impedance data were processed using ZView software (Scribner Associates, Inc., Southern Pines, NC, USA) and fitted to an electrically equivalent circuit. Differential-capacity plots (*dq/dV*) were acquired from 1.8 to 4.2 V at a 0.1 C-rate.

## 3. Results and Discussion

**Figure 2:**
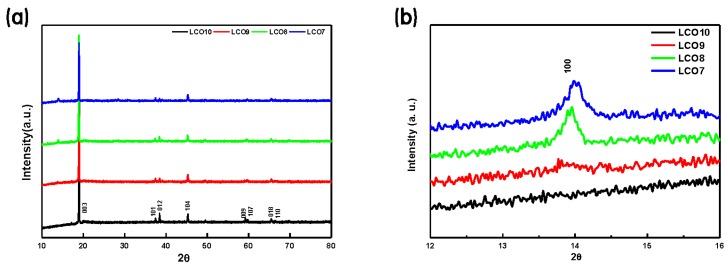
XRD patterns of (**a**) the LCO + LVO hybrid cathodes, and (**b**) the main peak of LVO between 12° and 16°.

The XRD patterns of the LCO + LVO hybrid cathodes indicated the effects of mechanical milling and are shown in Figure 2a,b. The major peak in each XRD profile (Figure 2a) corresponds to the LCO phase (JCPDS No. 50-0653), which was the largest component of each specimen. However, as shown in Figure 2b, a significant LVO peak (JCPDS No. 72-1193) was detected from 12° to 16° in the spectra of LCO9, LCO8, and LCO7, and the peak intensity increased with the LVO fraction. Thus, LCO and LVO existed independently in the hybrid cathodes, and no impurity phases were detected [14,32].

**Figure 3:**
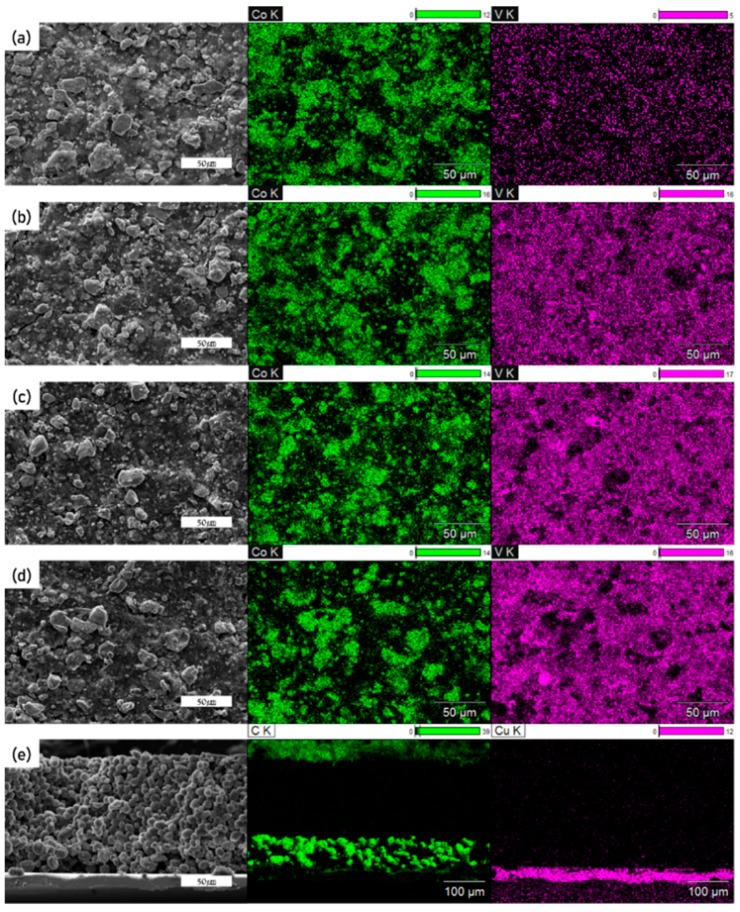
SEM images of the surfaces of the (**a**) LCO10, (**b**) LCO9, (**c**) LCO8, and (**d**) LCO7 electrodes, along with the EDX mappings showing the distributions of Co and V. (**e**) SEM image showing a side view of the graphite + LP hybrid anode.

Figure 3a–d presents SEM images of the surface morphologies of the LCO + LVO hybrid cathodes and EDX mappings indicating the distribution of Co and V. The LCO phase primarily existed in the form of spheroidal particles, and its content depended on the ratio of the two active materials. Although the LCO particles appeared to be larger than the LVO particles, the EDX analysis confirmed the presence of V and a uniform distribution of LVO. Therefore, LCO and LVO were well-distributed in the hybrid cathodes and mixed without any degradation or cross-reactions [14,15,16,17,18]. Figure 3e presents a side view of the anode, which was composed of Cu foil, graphite, and LP. The individual layers were distinguished through EDX mapping, with the LP layer between the green layers at the middle of Figure 3e. Because Li was not detectable through EDX mapping, it was represented using carbon tape at the top of the electrode. This layer, which had a well-ordered and clear appearance, was relatively thick despite its low weight. This was due to the low density of Li. These measurements indicate the successful preparation of the graphite + LP hybrid anode with a uniform LP layer and good contact between the materials.

**Figure 4:**
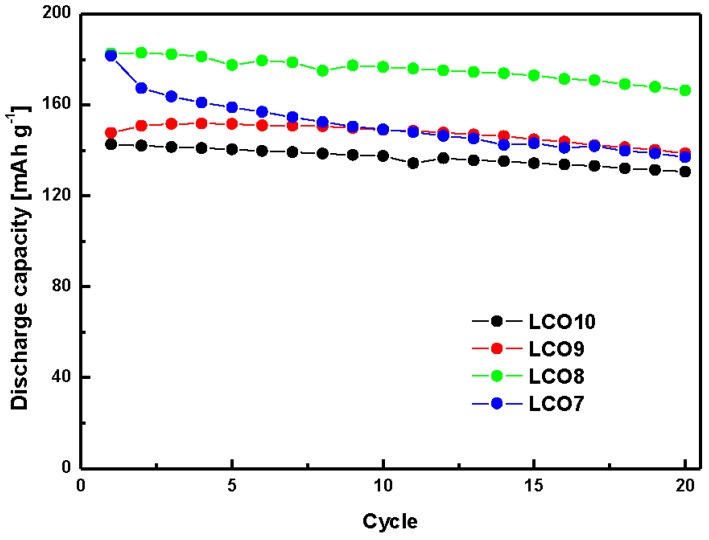
Cycle performances of the prepared LCO + LVO hybrid cathodes.

Figure 4 and Figure S1 show the cycle performance (0.1 C-rate) and rate capabilities of the hybrid cathodes, respectively. As the electrochemical properties of LCO and LVO had been previously characterized [14,15,16,17,18,32,33,34,35], the change in their capacities was determined for only the initial 20 cycles. The first-cycle discharge capacity of LCO8 was 182.69 mA h g^−1^, which was higher than those of the other cathodes. The initial capacity of LCO7 was similar to that of LCO8 but decreased rapidly with progressive cycling. The capacity retention rate of LCO7 was 75%, which was approximately 15% lower than those of the other cathodes. The capacity deterioration of LCO7 was likely due to its relatively high LVO content. Related results will be reported in the near future [36,37,38,39,40]. LCO8 exhibited a high discharge capacity and high retention as the number of cycles increased as well as a higher rate capability than the other hybrid cathodes at various current densities. In this study, the composition of LCO8 was considered optimal because it exhibited the highest discharge capacity and a retention rate of ≥90%. This composition yielded both a high retention rate of LCO and a high capacity of LVO. 

**Figure 5:**
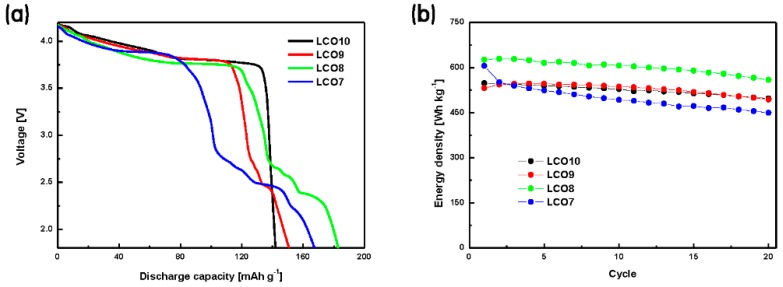
(**a**) Second-cycle discharge curves and (**b**) energy densities of the prepared LCO + LVO hybrid cathodes.

Figure 5a shows the second discharge curves for the LCO + LVO hybrid cathodes, from which the energy densities of the electrodes were calculated. In addition, the discharge capacity and nominal voltage were determined by calculating the energy density [41,42]. Table 2 presents the energy densities, discharge capacities, and nominal voltages of the prepared electrodes. Although the nominal voltage of LCO8 decreased by 10.48% with the addition of LVO as compared to that of LCO10 (3.844 V → 3.441 V), the voltage was sufficient to drive the cell. However, for the same reason, the overall capacity increased by 28.76% (142.03 mA h g^−1^ → 182.88 mA h g^−1^) because of the effect of LVO, which had approximately twice the typical reported capacity of LCO. Consequently, the energy density of LCO8 (i.e., 629.24 W h kg^−1^) was the highest among the prepared materials (15.25% higher than that of LCO10 (545.96 W h kg^−1^)). Figure S2 shows the first-cycle voltage curves of the prepared LCO + LVO hybrid cathodes. All the samples showed a coulombic efficiency greater than 94% despite the first cycle. Figure 5b shows the energy density over 20 cycles. The energy density of LCO8 remained high, similar to its cycle performance. Figure S3 shows an SEM image of the cathode after the cycling tests. Clearly, practically no change in the surface morphology of LCO8 occurred during the cycling test. However, side effects such as cracking began to appear in LCO7. Thus, LCO8 was the optimal condition.

**Figure 6:**
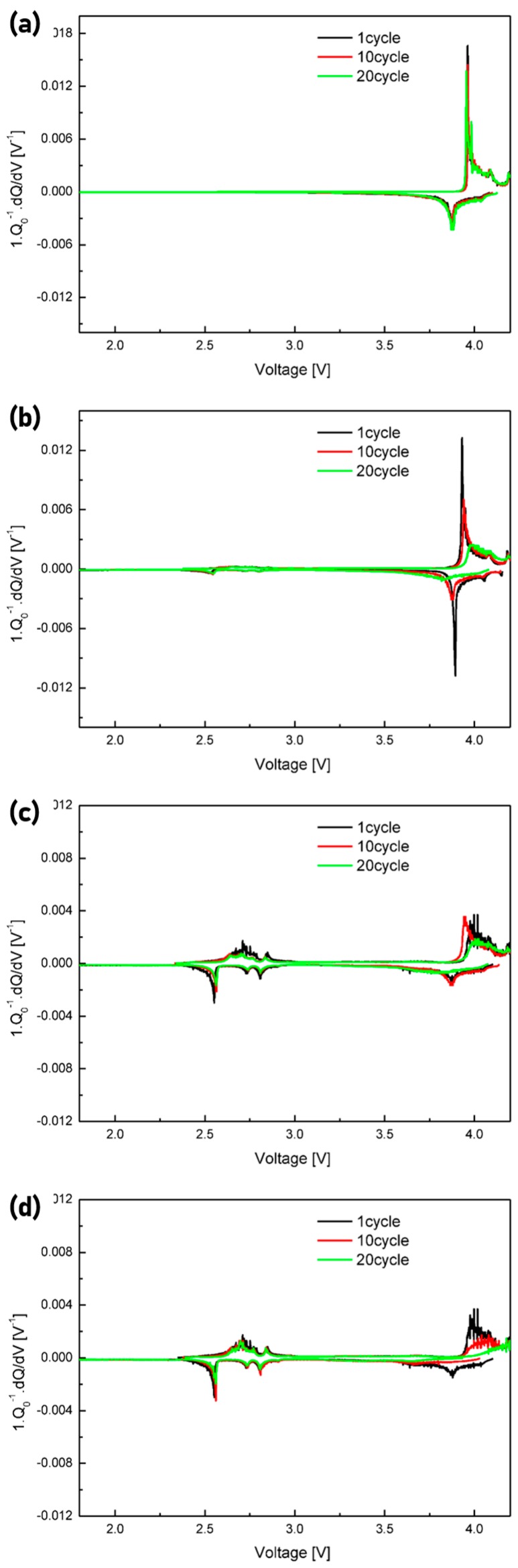
Differential-capacity curves of (**a**) LCO10, (**b**) LCO9, (**c**) LCO8, and (**d**) LCO7 for the 1st, 10th, and 20th cycles.

Figure 6a–d shows differential-capacity plots of the data acquired for the 1st, 10th, and 20th cycles for the prepared electrodes. As all four materials contained LCO, all the plots exhibited cathodic and anodic peaks related to the reactions of LCO. The main peaks corresponding to the first-order LCO transition (LiCoO_2_ → Li_0.8_CoO_2_) were detected at 3.87 V (cathodic reaction) and 3.95 V (anodic reaction). The minor peaks corresponding to the structural transition (hexagonal → monoclinic) were observed at ˃4 V [43]. The LVO reactions were related to the discharge mechanism (i.e., LP attached to the graphite electrode surface as a reservoir, intercalating with the LVO layer). The peaks attributed to the single-phase reaction and two-phase transition (i.e., Li_3_V_3_O_8_ → Li_4_V_3_O_8_) were observed at approximately 2.6–2.8 and 2.36 V, respectively, in the plots for LCO9, LCO8, and LCO7 [40]. These plots reveal that LCO and LVO reacted in different voltage ranges and existed independently in the hybrid cathodes.

**Figure 7:**
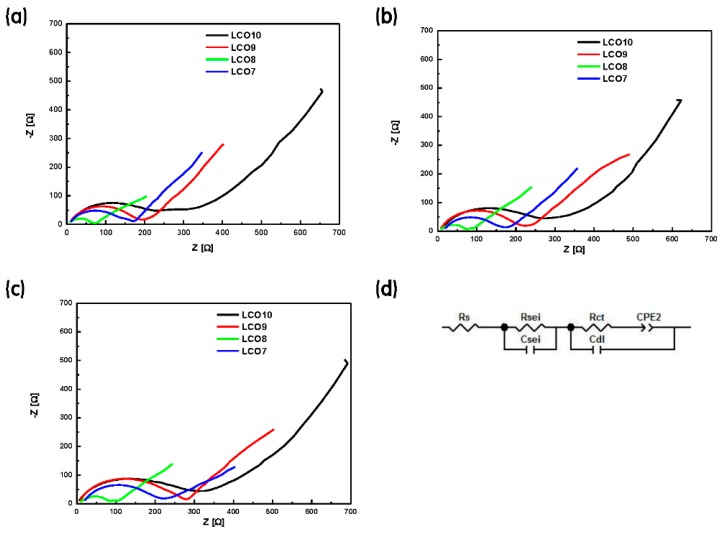
Impedance data for the LCO10, LCO9, LCO8, and LCO7 battery systems obtained from the (**a**) 1st, (**b**) 10th, and (**c**) 20th cycles. (**d**) Randles equivalent circuit used to fit the impedance data.

Figure 7a–c shows the impedance analysis data obtained during the 1st, 10th, and 20th cycles, respectively. Figure 7d shows the Randles equivalent circuit that was used to model the electrochemical reaction occurring on the surfaces of the LCO + LVO hybrid cathodes. Here, *R_s_* represents the sum of the ohmic resistances of the electrode and electrolyte; *R_sei_* and *C_sei_* represent the resistance and capacitance, respectively, of the solid–electrolyte interface; and *R_ct_* and *C_dl_* represent the charge-transfer resistance and double-layer capacitance, respectively, and are connected in parallel in the Randles circuit. *R_ct_* is a major indicator of electrochemical performance because it depends on the electrical conductivity, crystal structure, interparticle contacts, and surface conditions of the electrode. The constant-phase element (CPE2) is related to the *R_ct_* value and is indicated by the small semicircle in the corresponding Nyquist plot. Table 3 presents the *R_s_*, *R_sei_*, and *R_ct_* values that were determined by fitting the experimental impedance data with the equivalent circuit [44,45,46,47]. The *R_ct_* value and its variation were the smallest for LCO8, even after continued cycling. This indicates that LCO8 exhibited low polarization and rapid Li-ion migration, with negligible resistance between the electrode surface and electrolyte. Therefore, LCO8 exhibited higher electrical conductivity, stability, and electrochemical performance than the other cells [48,49].

## 4. Conclusions

In this study, a novel battery system consisting of an LCO + LVO hybrid cathode and graphite + LP hybrid anode was developed to increase the discharge capacity and energy density of LIBs. The LCO + LVO hybrid cathode was easily synthesized without sacrificing the inherent characteristics of the components. LCO8, which was composed of 80 wt% LCO and 20 wt% LVO, exhibited the best electrochemical performance (energy density of 629.24 wh kg^−1^ and discharge capacity of 182.88 mAh g^−1^). This was likely due to the synergistic effects between LCO and LVO. However, because excess LVO had negative side effects, the optimal content was limited to 20 wt%. Under the conditions of this study, the morphology of the electrode was maintained even after cycling, and the electrical conductivity remained high. The thickness of the anode, which was reduced because of the use of the LCO + LVO hybrid cathode, is expected to increase with improved energy density. The hybrid cathode could be further improved by developing methods to overcome the problems associated with LVO. Additional experiments to fabricate a hybrid cathode with other cathode materials (NCM, LFP, etc.) or to conduct electrochemical measurements at different temperatures are also necessary. Thus, this study provides novel insights into the development of high-energy-density LIBs.

## Figures and Tables

**Table 1:** Mass percentages of the components of the cathode and anode.

	Hybrid Cathode (wt%)		Hybrid Anode (wt%)		
	LCO	LVO	Graphite (g)	LP (g)	Graphite + LP (g)
**LCO10**	100	0	100 (0.737)	0 (0)	0.737
**LCO9**	90	10	88 (0.663)	12 (0.007)	0.670
**LCO8**	80	20	78 (0.589)	22 (0.015)	0.604
**LCO7**	70	30	70 (0.516)	30 (0.022)	0.537

**Table 2:** Energy densities, discharge capacities, and nominal voltages of the prepared electrodes.

	LCO10	LCO9	LCO8	LCO7
Energy density [W h kg^−1^]	545.96	543.84	629.24	551.49
Energy density [W h L^−1^]	2686.12	2578.89	2871.85	2418.84
Discharge capacity [mA h g^−1^]	142.03	150.84	182.88	167.35
Nominal voltage [V]	3.844	3.605	3.441	3.295

**Table 3:** *R*_s_, *R*_sei_, and *R*_ct_ values determined by fitting the impedance values with the Randles equivalent circuit in Figure 7d.

**LCO10**	**Cycle**			**LCO9**	**Cycle**		
	**1st**	**10th**	**20th**		**1st**	**10th**	**20th**
*R_s_* [Ω]	11.99	13.56	6.04	*R_s_* [Ω]	9.58	8.42	8.19
*R_sei_* [Ω]	62.48	73.45	74.01	*R_sei_* [Ω]	49.37	49.34	70.05
*R_ct_* [Ω]	138.50	154.90	179.60	*R_ct_* [Ω]	120.40	142.90	171.60
**LCO8**	**Cycle**			**LCO7**	**Cycle**		
	**1st**	**10th**	**20th**		**1st**	**10th**	**20th**
*R_s_* [Ω]	6.23	6.51	8.38	*R_s_* [Ω]	11.46	20.01	20.09
*R_sei_* [Ω]	21.41	21.83	20.76	*R_sei_* [Ω]	74.54	61.02	44.56
*R_ct_* [Ω]	35.90	42.66	51.16	*R_ct_* [Ω]	70.26	76.85	115.90

## Supplementary Materials

The following are available online at https://www.mdpi.com/1996-1944/12/12/2025/s1, Figure S1: Rate capabilities of the prepared LCO + LVO hybrid cathodes, Figure S2: First-cycle voltage curves of the prepared LCO + LVO hybrid cathodes, Figure S3: SEM images of the (a) LCO10, (b) LCO9, (c) LCO8, and (d) LCO7 electrode surfaces after cycling.
