# Supplementary material for: Energy-Density Improvement in Li-Ion Rechargeable Batteries Based on LiCoO2 + LiV3O8 and Graphite + Li-Metal Hybrid Electrodes
Source: Materials (Basel). 2019 Jun 24;12(12):2025. doi: 10.3390/ma12122025 (PMC6631142; doi:10.3390/ma12122025)
Supplement: Supplementary file 1 [file materials-12-02025-s001.pdf]

materials

Supplementary file

Energy-Density Improvement in Li-Ion Rechargeable Batteries Based on $LiCoO_2$ + LiV_3O_8 and Graphite + Li-Metal Hybrid Electrodes

Ki Yoon Bae, Sung Ho Cho, Byung Hyuk Kim, Byung Dae Son and Woo Young Yoon*

Department of Materials Science and Engineering, Korea University, 1, 5Ga Anam-dong, Sungbuk-gu, Seoul 136-701, Republic of Korea

* Correspondence: Tel: +82 2 3290 3274, E-mail: wyyoon@korea.ac.kr

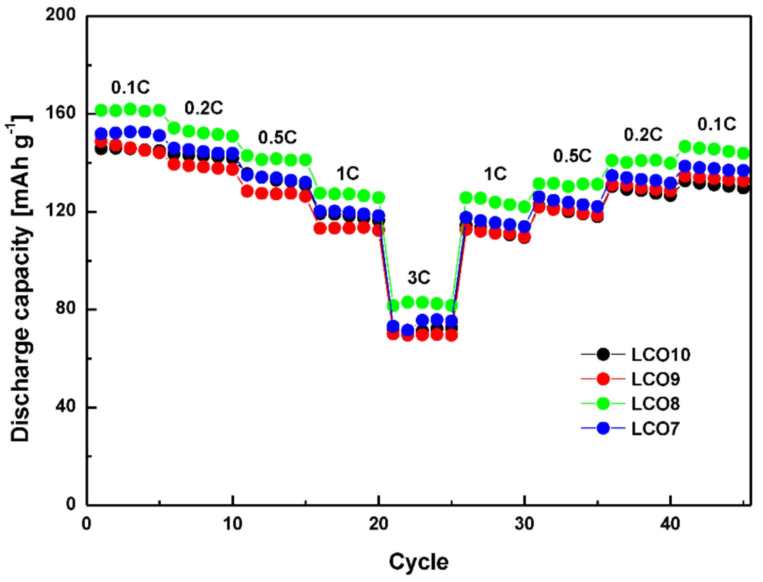

Figure 1. Rate capabilities of the prepared LCO + LVO hybrid cathodes.

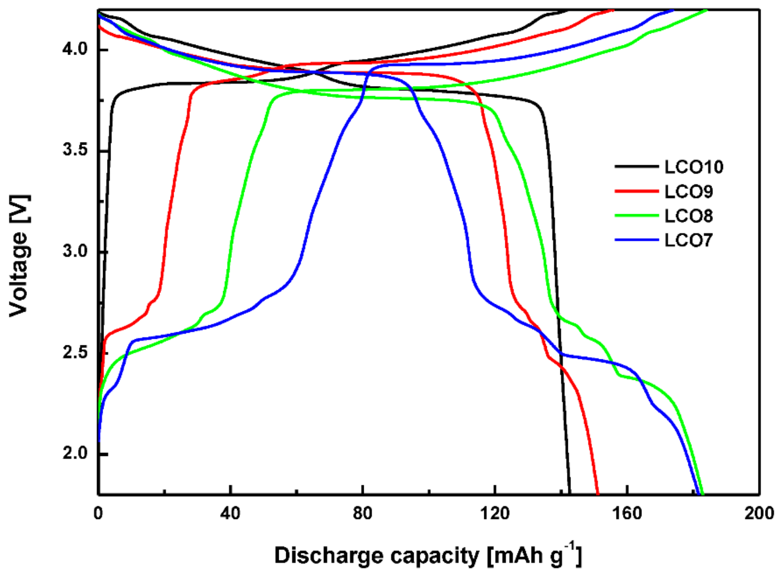

Figure 2. First-cycle voltage curves of the prepared LCO + LVO hybrid cathodes.

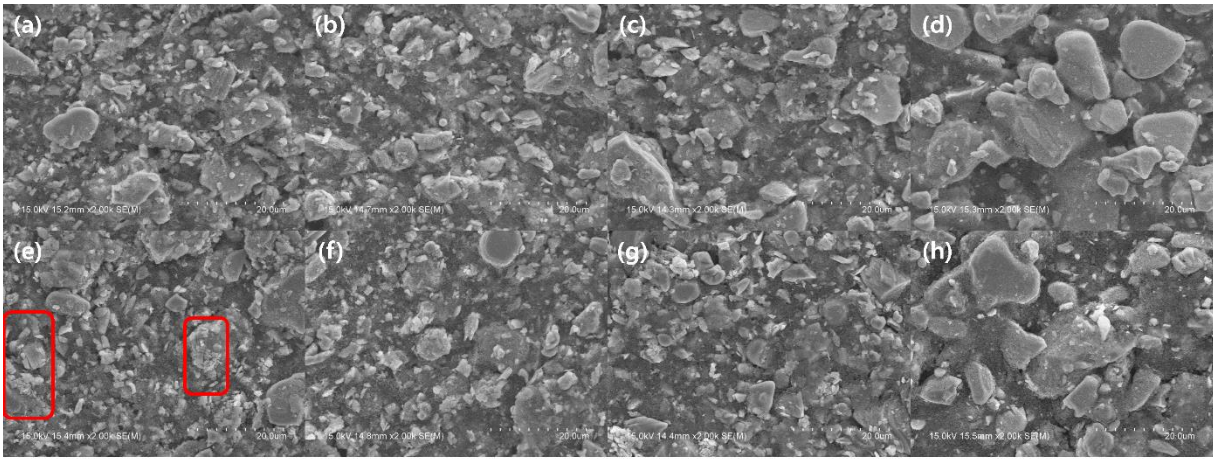

Figure 3. SEM images of the (a) LCO10, (b) LCO9, (c) LCO8, and (d) LCO7 electrode surfaces after cycling.
